# Dual PPAR **α**/**γ** Agonism Normalizes Lipoprotein Profile of Renal Dyslipidemia

**DOI:** 10.1155/2013/391628

**Published:** 2013-03-28

**Authors:** O. Samuelsson, P. O. Attman, I. Gause-Nilsson, M. K. Svensson, P. Alaupovic

**Affiliations:** ^1^Department of Nephrology, Sahlgrenska University Hospital, 41345 Göteborg, Sweden; ^2^AstraZeneca Research & Development, 43183 Mölndal, Sweden; ^3^Lipid and Lipoprotein Laboratory, Oklahoma Medical Research Foundation, Oklahoma City, OK, USA

## Abstract

Chronic kidney disease (CKD) is characterised by specific lipoprotein abnormalities and insulin resistance. Dual activation of the peroxisome proliferators-activated receptors (PPAR) **α** and **γ** can significantly improve insulin sensitivity. The aim of the study was to investigate the effects of a dual PPAR **α**/**γ** agonist on lipoprotein abnormalities in patients with CKD. One mg of the dual PPAR **α**/**γ** agonist tesaglitazar was given once daily during six weeks to CKD patients, and to healthy subjects. Plasma lipids, apolipoproteins (apo) and discrete lipoprotein subclasses were measured at baseline and end of treatment. In the CKD patients apoA-I increased significantly by 9%, and apoB decreased by 18%. There was an increase of apoC-III in HDL by 30%, and a parallel decrease of apoC-III in VLDL + LDL by 13%. Both the apoB-containing cholesterol-rich and the triglyceride-rich subclasses decreased significantly. With the exception of ApoC-III,all plasma lipids apolipoproteins and lipoprotein subclasses were reduced by treatment down to similar levels as the baseline levels of a healthy group of reference subjects. This study suggests that by improving insulin sensitivity a dual PPAR **α**/**γ** agonist has the potential to normalise most of the lipoprotein abnormalities in patients with CKD.

## 1. Introduction

Chronic renal insufficiency is characterized by specific lipoprotein abnormalities [1–3], insulin resistance, and accelerated cardiovascular disease (CVD) [4–6]. The renal dyslipidemia shares many features with the alterations of the lipoprotein metabolism found in patients with insulin resistance [7]. Hence, reduction of insulin resistance in chronic renal insufficiency could theoretically have positive effects on renal dyslipidemia and, consequently, also positive effects on CVD morbidity in patients with chronic kidney disease (CKD).

It is well documented that patients with chronic renal insufficiency as well as patients with diabetes mellitus are at high cardiovascular risk and that the characteristic lipoprotein abnormalities play an important role in atherogenesis [8, 9]. In a *post-hoc *analysis of the VA-HIT study the peroxisome proliferators-activated receptor (PPAR) *α* agonist gemfibrozil was shown to reduce cardiovascular morbidity in coronary patients with mild to moderate renal insufficiency [10]. Furthermore, in a *post-hoc* analysis of the PROactive trial the PPAR *γ*-agonist pioglitazone significantly reduced cardiovascular morbidity in type 2 diabetic patients with reduced renal function and documented macrovascular disease [11]. 

Tesaglitazar is a dual PPAR *α*/*γ* agonist previously in clinical development for the treatment of type 2 diabetes mellitus. It significantly improves insulin sensitivity [12]. However, the clinical development of tesaglitazar was discontinued when phase III studies indicated that the benefit-to-risk profile was unlikely to give patients a benefit over other currently available antidiabetic therapies [13–15]. 

The aim of the present analyses was to investigate the effects of a dual PPAR *α*/*γ* agonist on lipoprotein abnormalities in patients with CKD and various degrees of renal impairment.

## 2. Methods

### 2.1. Study Design and Study Groups

The study was an open-label study in two parallel groups. The primary aim of the study was to evaluate pharmacokinetics of tesaglitazar in patients with renal impairment [16]. A secondary, and prespecified, aim of the study was to analyze the pharmacodynamic effects on the lipoprotein metabolism in patients with nondiabetic, chronic kidney disease. 

One mg of tesaglitazar was given once daily during six weeks to patients with various degrees of renal impairment (renal impaired group) and to subjects with normal renal function (reference group). After completion of active drug treatment, the groups were followed for an additional three weeks. No dietary advice was given during the study and followup.

The aim was to include eight patients in each of the three groups of varying severities of renal impairment: mild renal impairment (GFR 51–80 mL/min × 1.73 m^2^ BSA), moderate renal impairment (GFR 31–50 mL/min × 1.73 m^2^ BSA), and severe renal impairment (GFR 10–30 mL/min × 1.73 m^2^ BSA). Twenty-three patients were finally included (*n* = 7, *n* = 8, and *n* = 8, resp.). All patients were nondiabetic, and no one had nephrotic-range proteinuria. Patients treated with any kind of pharmacological therapy that could interfere with the lipoprotein metabolism were excluded.

A group of 18 age- and sex-matched subjects with normal renal function was included as a reference group. One subject was withdrawn during the treatment period. Baseline characteristics of the groups are presented in Table 1.

The study was conducted according to the Declaration of Helsinki and in accordance with the Guideline of Good Clinical Practice and was approved by independent ethics committee. Signed informed consent was received from all subjects.

### 2.2. Procedures

Blood samples to determine plasma lipids, apolipoproteins, lipoproteins, and their sizes were drawn in all subjects at start and after six weeks, that is, at the end of active treatment. Plasma lipids were also determined after three-week followup of study drug. In addition, fasting plasma insulin and blood glucose were measured. All samples were taken after an overnight fast. To plasma samples for the lipoprotein measurements were added preservatives containing thimerosal and a protease inhibitor, *ε*-amino caproic acid, and they were immediately shipped by air to the Lipid and Lipoprotein Laboratory at the Oklahoma Medical Research Foundation in Oklahoma City, Oklahoma, USA, for analyses.

Total cholesterol and triglyceride concentrations were determined by enzymatic methods [17]. Apolipoproteins A-I, B, C-III, and E were measured by electroimmunoassays using monospecific antisera as previously described [18]. The distribution of apoC-III was determined by measuring apoC-III in heparin-Mn++ supernates (i.e., in HDL) and precipitates (in VLDL + LDL), and the apoC-III ratio (apoC-III in HDL: apoC-III in VLDL + LDL) was calculated [18].

Lipoproteins A-I and A-I : A-II and the major classes of apoB-containing lipoprotein families, cholesterol-rich Lp-B and triglyceride-rich Lp-B : C, Lp-B : C : E, and Lp-A-II : B : C : D : E were isolated by immunoaffinity chromatography and determined according to a previously described electroimmunoassay of apoB [18].

Lipoprotein particle size and concentration were analyzed by nuclear magnetic resonance (NMR) technique [19].

Glomerular filtration rate (GFR) was measured by plasma iohexol clearance at start and end of treatment and after three weeks of followup [20]. In patients with mild renal impairment and in subjects with normal renal function, blood samples were drawn for determination of plasma concentration of iohexol at 1, 2, and 4 hours after the injection of iohexol. In patients with moderate renal impairment samples were taken after 2, 3, and 5 hours, and in patients with severe renal impairment samples were taken at 5 and 24 hours after the injection of iohexol.

### 2.3. Statistical Analysis

Standard statistics were used to illustrate the salient features of data. Changes in metabolic and renal outcome variables from baseline were calculated for each group (renal impaired group and reference group) and analyzed in a linear model, using a fixed-effect analysis with the baseline value as covariate. A *P* value less than 0.05 (two-sided test) was regarded as statistically significant. 

## 3. Results

### 3.1. Plasma Lipids, Table 2

The plasma concentrations of triglycerides, total cholesterol, VLDL-cholesterol, and LDL-cholesterol were all significantly reduced by treatment with tesaglitazar in both the renal impaired group and the reference group. HDL-cholesterol increased significantly in subjects with renal impairment. The plasma lipids returned to baseline levels after three weeks of treatment.

### 3.2. Apolipoproteins, Table 3

In the subjects with renal impairment apoA-I increased significantly by 9%, and apoB decreased by 18%. The total plasma concentration of ApoC-III did not change. However, there was a significant change in the apolipoprotein content of apoC-III, that is, an increase in apoC-III in HDL by 30% and a parallel decrease in apoC-III in VLDL + LDL by 13% in subjects with renal impairment. This resulted in a significant increase in the apoC-III ratio by 55%. Although the level of apoC-III in VLDL + LDL also increased in the reference group, the apoC-III ratio did not change significantly. The total plasma concentration of apoE was unaltered in both groups.

### 3.3. Lipoprotein Subclasses (Lp), Tables 4 and 5

Lp A-I and A-I : A-II particles increased, and the apoB-containing cholesterol-rich subclass Lp-B and the triglyceride-rich subclasses LP-B : E + LP-B : C : E + LP-A-II : B : C : D : E decreased significantly (−17% and −32%, resp.) in the patients with CKD. The decrease of the apoB-containing triglyceride-rich lipoprotein subclasses was due to a significant decrease in the individual Lp-B : C (−26%) at the Lp-B : E + Lp-B : C : E (−28%), whereas no significant changes were observed in the Lp-A-II : B : C : D : E subclass concentrations. In the reference group there was a significant reduction of 13% in Lp-B, whereas the reduction of 26% of apoB-containing triglyceride-rich subclasses did not reach statistical significance (95% CI: −46%, +2%). 

The effect of tesaglitazar on apoB-containing lipoproteins increased with reduced renal function (Table 5). Lp-B, Lp-B : C and Lp-B : C : E were all reduced down to the same level as in the reference group at baseline before treatment; that is, a normalisation was observed after 6 weeks of treatment (due to small subject numbers in each subgroup no statistical inference test was performed).

### 3.4. Lipoprotein Particle Size

The lipoprotein particle size did not change during treatment. The LDL diameter was 21.0 (range 19.0–22.0) nm at baseline and 21.2 (range 20.1–22.0) after six weeks in the renal impaired group. The corresponding LDL diameters were 21.6 (range 20.9–22.0) and 21.6 (range 21.0–22.0), respectively, in the reference group. 

### 3.5. Fasting Insulin and Fasting Plasma Glucose

After 6 weeks of treatment, fasting insulin was reduced by 40% in the group with CKD (*P* < 0.01) and by 32% in the reference group. Fasting plasma glucose levels were unaltered in both groups.

### 3.6. Renal Function, Table 6

Glomerular filtration rate decreased in both study groups. In subjects with normal renal function it decreased from 94.6 to 91.9 mL/min × 1.73 m^2^ body surface area (BSA) and in the CKD patients from 43.2 to 37.7 mL/min × 1.73 m^2^ BSA. This reduction in GFR of 13% in the CKD patients was of the same magnitude in all three subgroups of mild, moderate, and severe renal impairment, respectively. With one exception GFR returned to the baseline value three weeks after the tesaglitazar treatment was stopped.

### 3.7. Safety Data and Adverse Events

Ten subjects in the renal impaired group reported a total of 15 adverse events. One was a gastrointestinal bleeding episode requiring hospitalisation, whereas the remaining 14 adverse events were mild. Twelve subjects in the reference group reported a total of 27 adverse events. One subject in the reference group was withdrawn from active treatment due to a herpes zoster infection. The other reported adverse events were all mild.

## 4. Discussion

The main finding of this study was that treatment with a dual PPAR *α*/*γ* agonist during six weeks was able to reverse and normalise most of the characteristic lipoprotein abnormalities of chronic renal impairment.

Since the primary purpose of the study was to evaluate the pharmacokinetics of the dual PPAR *α*/*γ* agonist tesaglitazar in subjects with renal impairment, the study was designed as an open-label study of two groups which both received active treatment. Thus, a placebo-controlled group was not included. The results of the pharmacokinetic study have been published elsewhere [16]. The lack of a randomised control group is of course a limitation of the study in analysing the pharmacodynamic effects of the drug. However, the effects on plasma lipids, insulin, and glucose were analyzed prior to treatment, at end of treatment, and also after three weeks of treatment. These data clearly show that the changes observed during active treatment almost returned to baseline levels after three weeks of withdrawal of the drug. Furthermore, the effect of treatment on all lipoprotein variables in the renal impaired group could be compared with baseline data in a reference group of age-sex matched subjects with normal renal function. Therefore, the changes that were observed during six weeks of treatment are most likely an effect by the treatment per se and similar effects have also been shown previously in other studies [21].

It is well documented that there is a typical accumulation of apoB-containing triglyceride-rich lipoproteins early in mild to moderate renal impairment, which is first detected in specific alterations in the apolipoprotein profile [1, 2]. With more advanced renal failure also plasma lipids are altered with a lipoprotein pattern similar, but not identical, to that of patients with insulin resistance [1–3]. Since chronic renal disease is an insulin-resistant state [4–6], any kind of intervention that can improve insulin sensitivity in subjects with renal impairment has a theoretical potential to normalise renal dyslipidemia.

**Figure 1:**
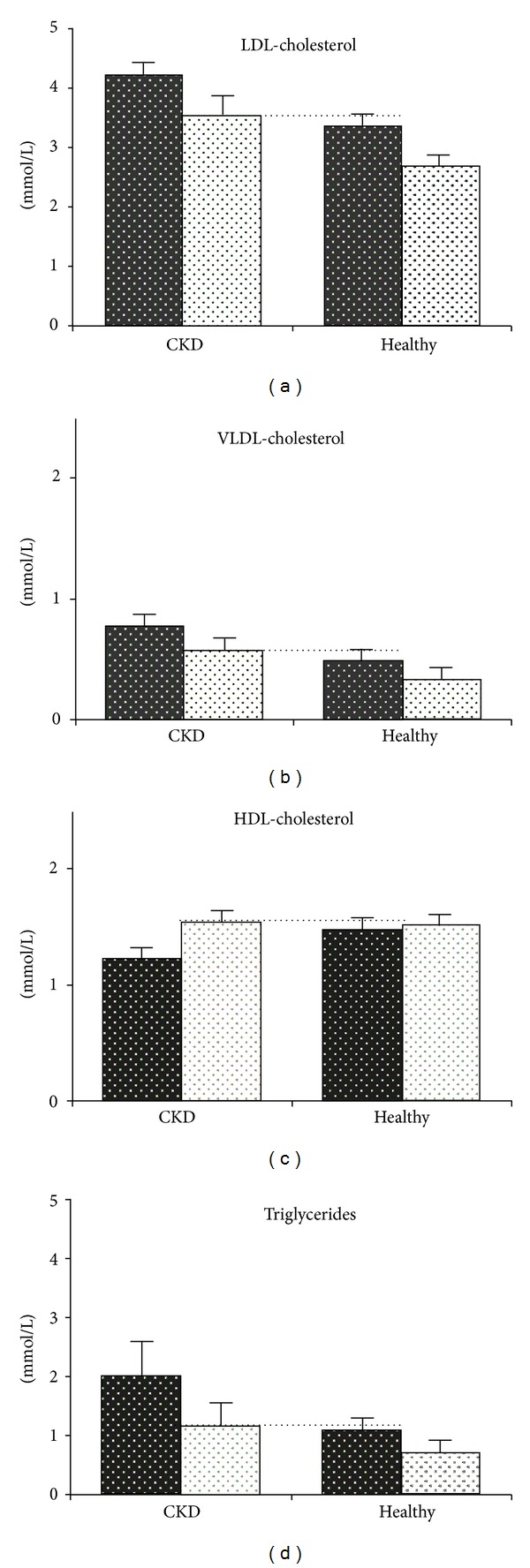
Plasma lipid concentrations at baseline and after six-week treatment with 1 mg tesaglitazar o.d. (mean and standard error of the mean).

All plasma lipids were significantly, and positively, altered in the patients with renal impairment. In fact, the lipid profile in the renal impaired group was normalised after six-week treatment when compared with the baseline profile of the subjects with normal renal function, illustrated in Figure 1. Also the plasma concentrations of apolipoproteins B and A-I, as well as their ratios (apoB/apo A-I), were normalised after treatment in comparison to the baseline levels in the reference group. The same effects of tesaglitazar were also observed in the reference group of subjects with normal renal function in whom HDL-cholesterol was further increased and the other plasma lipids were decreased to even lower levels after treatment. Similar qualitative changes were observed in the concentrations of plasma apolipoproteins.

In accordance with the increased plasma concentrations of apoA-I the plasma levels of the Lp-A-I and the Lp-A-I : A-II subclasses were significantly increased. The plasma concentrations of the atherogenic apoB-containing cholesterol-rich subclass, Lp-B, and the atherogenic apoB-containing triglyceride-rich subclasses carrying also apoC-III, that is, Lp-B : C and Lp-B : C : E, were reduced to the same levels seen at baseline in the reference group. This differs from previous findings by our group in the same category of subjects in which we found that fluvastatin treatment had a good effect on Lp-B but was less effective in reducing apoB-containing triglyceride-rich subclasses carrying also apoC-III [18], whereas the opposite pattern was observed in the controlled study of the PPAR *α* agonist gemfibrozil [22].

Although the elevated total plasma concentration of apoC-III was not affected by treatment with the dual PPAR *α*/*γ* agonist tesaglitazar, the apoC-III ratio was normalized. This is of great importance since it indicates a markedly increased catabolic rate of triglyceride-rich lipoproteins with a facilitated transfer of apoC-III from VLDL and IDL to HDL [23], which is in full accordance with the observed reduction of Lp-B : C and Lp-B : C : E. Furthermore, the reduction of fasting insulin levels without any alteration in fasting plasma glucose both in the renal impaired group and the reference group indicates that insulin sensitivity was enhanced by treatment with the dual PPAR *α*/*γ* agonist. One may speculate that the improved insulin sensitivity could potentially be due to the transfer of apoC-III from lipoproteins in VLDL- and LDL-density classes to lipoprotein subclasses in HDL, since it has been demonstrated that the plasma concentration of apoC-III bound to apoB-containing lipoproteins strongly correlates with the degree of insulin resistance in subjects with the metabolic syndrome [24].

Chronic renal disease is associated with a high cardiovascular risk [8], and it is clearly documented that cardiovascular morbidity increases gradually with reduced renal function [8, 25]. Both traditional and nontraditional risk factors probably play important roles in the development of atherosclerosis in chronic renal impairment [8, 26]. In parallel to the high cardiovascular risk of patients with type 2 diabetes, which to a large extent is due to alterations of lipoprotein metabolism [27], it is plausible that renal dyslipidemia is also of importance for the development of atherosclerosis in patients with CKD. 

The changes in the lipoprotein profile observed in this study by the dual PPAR *α*/*γ* agonist should be beneficial from a cardiovascular point of view. Recent documentation has established that the apoB plasma concentration and the apoB/ApoA-I ratio are stronger predictors of cardiovascular complications than the plasma lipid levels even in subjects with low and normal LDL-cholesterol [28, 29]. Thus, their normalisation by treatment could reduce the overall cardiovascular risk in patients with CKD. 

Whether the unaffected high plasma concentration of the apoC-III still implies an increased risk of atherosclerosis remains to be clarified. Studies on nonrenal patients have shown that apoC-III is an independent risk factor for coronary heart disease [30–32]. A detailed analysis of the CARE trial showed that it was the apoC-III concentration in VLDL and LDL that was a strong predictor of coronary events [30]. Moreover, Kawakami et al. have shown in a series of experiments that apoC-III in VLDL activates peripheral monocytes and vascular endothelial cells with increased expression of adhesion molecules, induces insulin resistance in endothelial cells, and causes endothelial dysfunction [33]. However, they also showed that isolated apoC-III and apoC-III in HDL had an adverse effect on HDL particles with regard to monocyte adhesion. Thus, apoC-III *per se* may be directly involved in atherogenesis. Therefore, effective therapeutic approaches that are able to regulate apoC-III metabolism may be of particular importance in this category of high-risk renal patients.

The clinical development of tesaglitazar was discontinued in 2006 when data from a 24-week randomised, controlled study of patients with type 2 diabetes mellitus clearly showed that the previously observed increase in serum creatinine concentrations was due to a true reduction in GFR and not to a reduction in the tubular secretion of creatinine [15]. The decrease in GFR was also observed in the present study of nondiabetic CKD patients. Similar to the observation in the present study, GFR returned to baseline levels after 4 to 12 weeks in the study of type 2 diabetics (15%). Thus, at least in the short term this negative effect on renal function seems to be reversible.

In conclusion, this study suggests that by improving insulin sensitivity a dual PPAR *α*/*γ* agonist has the potential to reverse and normalise most of the lipoprotein abnormalities in patients with CKD and chronic renal failure.

## Figures and Tables

**Table 1:** Baseline characteristics of patient and reference groups. Mean and standard deviation (in parenthesis).

	CKD patients	Healthy reference group
Age (years)	54.9 (11.9)	55.6 (10.3)
Male/female (*n*)	17/6	13/4
Body mass index (kg/m^2^)	27.1 (3.9)	25.0 (2.4)
S-creatinine (*µ*mol/L)	201 (66)	71 (11)
Glomerular filtration rate (mL/min × 1.73 m^2^ BSA)	43.2 (24.0)	94.6 (13.2)
Blood pressure (mm Hg)	140 (17)/81 (10)	135 (14)/81 (7)

**Table 2:** Plasma lipid levels (mmol/L) in patients with chronic renal insufficiency (*n* = 23) and healthy reference subjects (*n* = 17) at start and end of treatment with 1 mg tesaglitazar o.d. Mean and standard deviation (in parenthesis).

	CKD patients				Healthy reference group			
	At entry	After 6 weeks	Estimated change	After 3 weeks of drug	At entry	After 6 weeks	Estimated change	After 3 weeks of drug
Total cholesterol	6.4 (1.3)	5.7 (1.3)	−12%***	6.3 (1.2)	5.3 (0.7)	4.6 (0.5)	−14%***	5.0 (0.6)
Triglycerides	2.0 (1.9)	1.2 (0.9)	−40%***	1.7 (1.1)	1.1 (0.4)	0.7 (0.3)	−34%***	1.1 (0.4)
VLDL-cholesterol	0.8 (0.4)	0.5 (0.3)	−40%***	0.8 (0.5)	0.5 (0.2)	0.3 (0.1)	−34%***	0.5 (0.2)
LDL-cholesterol	4.2 (0.81)	3.5 (1.2)	−21%***	4.2 (1.0)	3.4 (0.7)	2.7 (0.6)	−20%**	3.1 (0.6)
HDL-cholesterol	1.2 (0.2)	1.6 (0.4)	+24%***	1.4 (0.3)	1.5 (0.4)	1.5 (0.4)	+2%	1.4 (0.3)

**P < 0.01 and ***P < 0.001 with group comparison between baseline and after 6 weeks of treatment.

**Table 3:** Plasma apolipoprotein concentrations levels (mg/dL) in patients with chronic renal insufficiency (*n* = 23) and healthy reference subjects (*n* = 17) at start and end of treatment with 1 mg tesaglitazar o.d. Mean, standard deviation, and 95% confidence interval (in parenthesis).

	CKD patients			Healthy reference group		
	At entry	After 6 weeks	Estimated change	At entry	After 6 weeks	Estimated change
Apo A-I	141 (16)	153 (21)	+9%**	145 (23)	154 (20)	+7%
Apo B	126 (34)	104 (31)	−18%***	105 (16)	87 (17)	−17%***
Apo B/Apo A-I	0.9 (0.3)	0.7 (0.3)	−24%***	0.7 (0.2)	0.6 (0.1)	−22%***
Apo C-III	16.6 (5.4)	17.4 (3.9)	+6%	11.4 (2.4)	11.1 (2.5)	−3%
Apo C-III-HS	9.1 (2.3)	11.4 (3.8)	+30%***	8.3 (2.5)	8.1 (2.3)	+2%
Apo C-III-HP	6.8 (3.0)	5.7 (2.6)	−13%**	3.6 (1.0)	3.2 (1.2)	−14%**
Apo C-III ratio	1.5 (0.7)	2.5 (1.6)	+55%**	2.3 (0.9)	2.9 (1.6)	+19%
Apo A-I/Apo C-III	9.0 (2.0)	9.2 (2.0)	+9%	13.2 (2.7)	14.3 (2.5)	+2%
Apo E	7.8 (2.9)	8.2 (2.6)	+5%	5.5 (1.2)	6.0 (1.8)	+8%

**P < 0.01 and ***P < 0.001 with group comparison between baseline and after 6 weeks of treatment.

**Table 4:** Plasma concentrations of lipoprotein subclasses (mg/dL) in patients with chronic renal insufficiency (*n* = 23) and healthy reference subjects (*n* = 17) at start and end of treatment with 1 mg tesaglitazar o.d. Mean and standard (in parenthesis).

	CKD patients			Healthy reference group		
	At entry	After 6 weeks	Estimated change	At entry	After 6 weeks	Estimated change
Lp-A-I	36 (5)	40 (6)	+11%**	37 (7)	37 (7)	−1%
Lp-A-I : A-II	105 (12)	114 (17)	+8%**	108 (20)	117 (15)	+10%
Lp-B	79 (18)	67 (19)	−17%***	67 (11)	58 (10)	−13%**
Lp-B : C	10 (8)	7 (3)	−26%*	6 (2)	5 (2)	−19%
Lp-B : E + Lp-B : C : E	20 (8)	14 (6)	−28%**	13 (6)	10 (4)	−11%
Lp-A-II : B : C : D : E	17 (7)	16 (9)	−9%	20 (9)	15 (8)	−29%*

*P < 0.05, **P < 0.01, and ***P < 0.001 with group comparison between baseline and after 6 weeks of treatment.

**Table 5:** Plasma concentrations of Apo-B-containing lipoprotein particles (mg/dL) in patients with various degrees of chronic renal insufficiency (mild; *n* = 7, moderate; *n* = 8, severe; *n* = 8) and healthy reference subjects (*n* = 17) at start and end of treatment with 1 mg tesaglitazar o.d. Mean and standard deviation (in parenthesis).

	At entry	After 6 weeks	At entry	After 6 weeks
	Lp-B		Lp-B : C	

Reference group	67 (12)	58 (10)	6 (2)	5 (2)
Mild CRF	66 (9)	59 (13)	6 (1)	7 (2)
Moderate CRF	85 (21)	70 (22)	9 (5)	6 (2)
Severe CRF	86 (17)	70 (20)	15 (12)	8 (4)

	Lp-B : E + Lp-B : C : E		Lp-A-II : B : C : D : E	

Reference group	13 (6)	10 (4)	20 (9)	15 (8)
Mild CRF	14 (5)	15 (3)	16 (6)	9 (3)
Moderate CRF	22 (7)	14 (5)	17 (6)	17 (7)
Severe CRF	24 (9)	14 (10)	16 (9)	20 (10)

**Table 6:** Glomerular filtration rate and serum creatinine at entry, after administration of 1 mg tesaglitazar for 6 weeks, and at followup 3 weeks after drug administration. Mean and standard deviation (in parenthesis).

	CKD patients			Healthy reference group		
	At entry	After 6 weeks	After 3 weeks of drug	At entry	After 6 weeks	After 3 weeks of drug
GFR (mL/min × 1.73 m^2^ BSA)	43.2 (24.0)	37.7 (21.1)	43.4 (28.3)	94.6 (13.2)	91.9 (15.5)	91.8 (10.4)
S-creatinine (*µ*mol/L)	201 (66)	249 (93)	226 (89)	71 (11)	79 (8)	72 (10)
